# Mediterranean Diet Adherence, Dietary Components, and Vision-Related Quality of Life in Type 2 Diabetes: A Cross-Sectional Study According to Diabetic Retinopathy Status

**DOI:** 10.3390/nu18121970

**Published:** 2026-06-18

**Authors:** Agostino Milluzzo, Andrea Maugeri, Martina Barchitta, Roberta Magnano San Lio, Daniela Rocca, Antonio Marino, Lucia Frittitta, Laura Sciacca, Antonella Agodi

**Affiliations:** 1 Department of Clinical and Experimental Medicine, University of Catania, Via Santa Sofia 89, 95123 Catania, Italy; agostino.milluzzo@unict.it (A.M.); lucia.frittitta@unict.it (L.F.); 2 Diabetes and Obesity Center, Garibaldi-Nesima Hospital, 95122 Catania, Italy; 3 Department of Medical and Surgical Sciences and Advanced Technologies “GF Ingrassia”, University of Catania, Via Santa Sofia 78, 95123 Catania, Italy; andrea.maugeri@unict.it (A.M.); martina.barchitta@unict.it (M.B.); robertamagnanosanlio@unict.it (R.M.S.L.); antonella.agodi@unict.it (A.A.); 4 Ophtalmology Unit, Garibaldi-Nesima Hospital, 95122 Catania, Italy; danielamariarocca@gmail.com (D.R.); amarino@arnasgaribaldi.it (A.M.); 5 Endocrinology, Garibaldi-Nesima Hospital, 95122 Catania, Italy

**Keywords:** type 2 diabetes, diabetic retinopathy, Mediterranean Diet Score, dietary components, legumes, micronutrients, vision-related quality of life, NEI-VFQ, cross-sectional study

## Abstract

**Background/Objectives:** Diabetic retinopathy (DR) is a major microvascular complication of type 2 diabetes (T2D) and a leading cause of visual impairment. The relationships among Mediterranean diet adherence, dietary components, DR, and vision-related quality of life remain incompletely defined. This cross-sectional study evaluated Mediterranean Diet Score (MDS) as the primary dietary endpoint, individual MDS components as secondary endpoints, and micronutrient intakes as exploratory endpoints. **Methods:** In this single-centre study, 129 subjects with long-standing T2D were classified as no DR (NDR; *n* = 85), non-proliferative DR (NPDR; *n* = 36), or proliferative DR (PDR; *n* = 8). Dietary intake was assessed using a food frequency questionnaire and vision-related quality of life using the NEI-VFQ-25. **Results:** Subjects with DR had longer diabetes duration than those without DR (18 vs. 16 years, *p* < 0.01). Overall MDS did not differ by DR status, indicating a null finding for the primary dietary endpoint. In secondary analyses, lower legume consumption was observed among participants with DR and was associated with higher odds of DR in multivariable models. Participants with PDR showed poorer vision-related quality of life, although this finding was limited by the small PDR subgroup and high NEI-VFQ-25 scores in other groups. Exploratory analyses suggested associations between selected micronutrient intakes and NEI-VFQ-25 domains. **Conclusions:** Overall Mediterranean diet adherence was not associated with DR status. Secondary and exploratory findings should be considered hypothesis-generating and require confirmation in prospective studies.

## 1. Introduction

Diabetes mellitus (DM) represents a major global public health challenge, with a continuously increasing prevalence and a substantial clinical and socio-economic burden [1]. Type 2 diabetes mellitus (T2D), accounting for more than 90% of all diabetes cases, is the main contributor to diabetes-related morbidity and mortality through the development of chronic vascular complications [2,3].

Diabetic retinopathy (DR) is one of the most frequent and disabling microvascular complications of diabetes and remains a leading cause of vision impairment among working-age adults. Currently, the prevalence of DR ranges from 25% to 40% in T2D, the lifetime risk of developing DR exceeds 50% in individuals with T2D, and its prevalence is expected to further increase in parallel with the global diabetes epidemic [4,5,6,7]. Because DR may remain clinically silent until advanced stages, early identification of individuals at higher risk and effective preventive strategies are essential to reduce vision loss and its related impact on quality of life.

Chronic hyperglycaemia and diabetes duration are the strongest established risk factors for the development and progression of DR [8,9,10]. However, glycaemic exposure alone explains only a limited proportion of the inter-individual variability in DR risk. Other cardiometabolic factors, including hypertension, dyslipidaemia, obesity, coexisting metabolic disorders and comorbidities, further contribute to retinal damage, yet together they account for less than 10–15% of the overall risk of DR [9,11,12,13,14].

Genetic susceptibility has been shown to influence the development and severity of DR, as supported by familial clustering, twin studies, and ethnic differences in disease prevalence [15,16,17]. More recently, epigenetic mechanisms have been proposed as potential mediators linking environmental exposures and metabolic control to retinal damage [18,19,20,21]. However, while these biological pathways provide a plausible framework for understanding DR heterogeneity, their clinical relevance and translational applicability remain under investigation. These observations suggest that additional modifiable determinants may influence susceptibility to DR and its clinical course.

Lifestyle factors, particularly dietary habits and physical activity, have emerged as potential modulators of diabetes-related complications [22,23,24,25]. Healthy dietary patterns may improve glycaemic control, reduce oxidative stress, attenuate chronic low-grade inflammation, and support endothelial function, all mechanisms involved in DR pathophysiology [26,27,28]. Among dietary models, the Mediterranean diet (MD) has received considerable attention because of its well-established cardiometabolic benefits. It is characterized by high intake of fruits, vegetables, whole grains, legumes, olive oil, and fish, and lower consumption of saturated fats and red meat.

Evidence linking MD adherence and DR remains heterogeneous. Some observational studies and randomized trials suggest that healthier dietary patterns or specific MD components may be associated with a lower incidence or slower progression of retinopathy [29,30,31]. Nutrients and food groups may influence pathways relevant to DR, including glycaemic control, oxidative stress, inflammation, and endothelial function [32,33,34,35].

Given the high prevalence of DR, its impact on visual function and quality of life, and the limited proportion of risk explained by traditional factors, identifying modifiable lifestyle determinants represents a key clinical priority. Therefore, the primary objective of this study was to evaluate whether overall adherence to the Mediterranean diet, assessed by the Mediterranean Diet Score (MDS), differed according to the presence of DR in individuals with T2D. Secondary objectives were to examine the association between individual MDS components and DR. Exploratory analyses evaluated selected micronutrient intakes and vision-related quality of life across DR stages.

## 2. Subjects and Methods

### 2.1. Study Design

This cross-sectional study enrolled 129 Caucasian subjects affected by T2D, recruited during routine outpatient visits at the Diabetes Centre of the Garibaldi-Nesima Medical Centre (Catania, Italy) between January and July 2021. Eligible participants were aged over 40 years, had long-standing T2D, and had glycated haemoglobin (HbA1c) values between 7.0% and 9.5% at the time of recruitment. Exclusion criteria included ocular diseases other than DR, immune system disorders, active malignancies, and systemic therapies in the three months preceding enrolment. The diagnosis of T2D was established according to the American Diabetes Association criteria [2].

The study was conducted in accordance with the Declaration of Helsinki. All participants provided written informed consent prior to enrolment, and the study protocol was approved by the Institutional Ethics Committee of Catania 2 (protocol number 601/CECT2).

### 2.2. Data Collection

At recruitment visit, clinical data were collected, including medical history, diabetes duration, current anti-hyperglycaemic treatment, diabetes-related microvascular and macrovascular complications, and comorbidities. Sociodemographic and lifestyle information was obtained by trained epidemiologists using a structured questionnaire. Educational level was classified as low (≤8 years of schooling) or high (>8 years). Employment status was categorised as employed or unemployed (including students and housewives), and smoking status as smoker or non-smoker (including former smokers).

Anthropometric measurements included body weight, height, body mass index (BMI), and waist circumference (WC). BMI was calculated as weight in kilograms divided by height in meters squared and classified as normal weight (18.5–24.9 kg/m^2^), overweight (25.0–29.9 kg/m^2^), or obese (≥30.0 kg/m^2^) [36]. Waist circumference was measured at the iliac crest and considered abnormal when >88 cm in women and >102 cm in men, according to the National Cholesterol Education Program Adult Treatment Panel III criteria [36]. Systolic and diastolic blood pressure were measured using standardised procedures.

Laboratory parameters included HbA1c, total cholesterol, high-density lipoprotein (HDL) cholesterol, triglycerides, low-density lipoprotein cholesterol (LDL) (calculated using the Friedewald formula when triglycerides were <400 mg/dL [37]), aspartate aminotransferase (AST), alanine aminotransferase (ALT), and serum creatinine. Estimated glomerular filtration rate (eGFR) was calculated using the Chronic Kidney Disease Epidemiology Collaboration equation [38]. Urinary albumin-to-creatinine ratio (UACR) was assessed on a random spot urine sample, and albuminuria was classified according to ADA criteria.

### 2.3. Ocular Assessment

#### 2.3.1. Instrumental Examinations

Based on retinal findings, participants were classified into three groups: subjects without diabetic retinopathy (NDR), subjects with non-proliferative diabetic retinopathy (NPDR), and subjects with proliferative diabetic retinopathy (PDR). All participants underwent a comprehensive ophthalmological examination, including assessment of visual acuity, intraocular pressure, retinal fundus photography, fluorescein angiography, and optical coherence tomography. Retinal images were independently and blindly evaluated by two experienced ophthalmologists and classified according to the International Clinical Disease Severity Scale for Diabetic Retinopathy [39,40].

#### 2.3.2. National Eye Institute Visual Function Questionnaire (NEI-VFQ 25)

To assess the vision-related quality of life (VRQoL) we used the 25-item National Eye Institute Visual Function Questionnaire (NEI-VFQ) [41,42]. The VFQ-25 takes approximately 10 min to administer to subjects and consists of a base set of 25 vision-targeted questions representing 11 vision-related constructs, plus an additional single-item general health-rating question. The VFQ-25 generates the following vision-targeted subscales: global vision rating, difficulty with near vision activities, difficulty with distance vision activities, limitations in social functioning due to vision, role limitations due to vision, dependency on others due to vision, mental health symptoms due to vision, driving difficulties, limitations with peripheral and colour vision, and ocular pain. In addition, the VFQ-25 contains an extra question about the general health rating.

To calculate the VFQ-25 score, we recoded the original values from the survey according to specific scoring rules so that a high score represents better visual functioning. Each item was converted to a 0 to 100 scale, in order that the scores represent the achieved percentage of the total possible score (e.g., a score of 50 represents 50% of the highest possible score). Thus, items within each sub-scale were averaged together to create the 12 sub-scale scores. To calculate the overall composite score for the VFQ-25, we averaged the vision-targeted subscale scores, excluding the general health-rating question.

### 2.4. Dietary Assessment

Dietary intake was recorded using a 95-item semi-quantitative Food Frequency Questionnaire (FFQ), which referred to dietary habits during the previous month and was adapted from a 46-item FFQ validated for the assessment of folate intake in Italian women of childbearing age [43]. During the interview, participants reported consumption frequency (12 categories ranging from “almost never” to “two or more times a day”) and portion size (small, medium, or large). Medium portion sizes were described using standard weights or volume measures commonly consumed in the Italian population, while small and large portions corresponded to approximately half and at least 1.5 times the medium portion, respectively. A photographic atlas was used to improve portion-size estimation and reduce misclassification. Food intakes were estimated by multiplying the reported frequency of consumption by the corresponding daily portion size for each FFQ item. Nutrient intakes were calculated by linking FFQ food items to nutrient-composition values from the United States Department of Agriculture (USDA) FoodData Central database and were subsequently adjusted for total energy intake using the residual method [44].

#### Mediterranean Diet Score

The adherence to MD was investigated by using the MDS, which refers to the ideal or poor consumption of nine food categories: fruits and nuts, vegetables, legumes, cereals, lipids, fish, dairy products, meat products, alcohol and unsaturated to saturated lipids ratio [45,46]. For vegetables, legumes, fruits and nuts, cereals, fish and unsaturated to saturated lipids ratio, subjects whose consumption was below or equal to the median value of the population were assigned the value of 0, otherwise the value of 1. For dairy and meat products, subjects whose consumption was below the median were assigned a value of 1, otherwise the value of 0. To subjects consuming 5 to 25 g of alcohol per day, a value of 1 was assigned. Finally, MDS ranged from 0 (non-adherence) to 9 (perfect adherence). The adherence was further categorized as follows: low (MDS: 0–3), medium (MDS: 4–6), or high adherence (MDS: 7–9) [47].

### 2.5. Statistical Analysis

Continuous variables were tested for normality using the Shapiro–Wilk test and are presented as median and interquartile range (IQR). Comparisons between two groups were performed using the Mann–Whitney U test, while comparisons across more than two groups were conducted using the Kruskal–Wallis test. Categorical variables are expressed as frequencies and percentages and were compared using the chi-square or Fisher’s exact test, as appropriate.

The primary endpoint was the difference in overall adherence to the Mediterranean diet, assessed using the MDS, between participants with and without DR. Secondary endpoints included the individual components of the MDS, in order to evaluate whether specific food groups contributing to the overall score were associated with DR status. Exploratory analyses were conducted to describe selected micronutrient intakes across DR stages, to assess vision-related quality of life using NEI-VFQ-25 scores, and to examine Spearman rank correlations between selected clinical or dietary variables and NEI-VFQ-25 domain scores. Because these exploratory analyses involved multiple comparisons and no formal multiplicity correction was applied, their results were interpreted as hypothesis-generating rather than confirmatory.

Multivariable logistic regression models were constructed to evaluate the association between low legume consumption and the presence of DR. Low legume consumption was defined as intake below or equal to the cohort-specific median, consistent with the MDS scoring approach. Given the limited number of DR cases, model complexity was restricted and covariates were selected a priori according to their relevance to DR and potential confounding. Model 1 was adjusted for age and sex. Model 2 was further adjusted for diabetes duration and HbA1c, which was included a priori because of its established role in the pathophysiology of DR. Model 3 additionally included insulin treatment as a marker of diabetes severity and treatment intensity. To reduce the risk of overfitting, no further covariates were included in the multivariable models. Results are reported as adjusted odds ratios (ORs) with 95% confidence intervals (CIs).

A two-sided *p* value < 0.05 was considered statistically significant. Statistical analyses were performed using IBM SPSS software (version 21.0).

#### Sample Size Calculation

The sample size was calculated based on the primary study endpoint, namely a between-group difference in the MDS. Assuming a mean difference of 2 points in the MDS between groups, a standard deviation of 5, an alpha level of 0.05, and a statistical power of 80%, a minimum of 98 subjects (32 with DR and 64 without DR) was required. Analyses of individual MDS components and micronutrients were considered secondary and exploratory, respectively.

## 3. Results

### 3.1. Study Population and Clinical Characteristics

One hundred twenty-nine participants with T2D were enrolled between January and July 2021, including 65 men (50.4%) and 64 women (49.6%). The main clinical and demographic characteristics of the study population are reported in Table 1. The median age at enrolment was 70 years (IQR 65–74), while the median age at T2D diagnosis was 53 years (IQR 46–58), corresponding to a median diabetes duration of 17 years (IQR 14–19). Only approximately one-tenth of participants had a normal body weight, whereas the majority were classified as overweight (41.9%) or obese (45.0%). An above-normal WC was observed in 80.6% of the study population. The median HbA1c level was 8% (IQR 7–8). Overall, 41.9% of subjects were treated with insulin, generally in combination with other oral or injectable glucose-lowering agents. In addition, lipid-lowering therapy was used by 76.7% of participants, antihypertensive drugs by 82.2%, and antiplatelet agents by 71.2%. Kidney-related variables were reported descriptively as eGFR and albuminuria. Microalbuminuria and macroalbuminuria were observed in 18.6% and 0.8% of participants, respectively. Regarding chronic macrovascular complications, 16.3% reported a history of coronary ischemic disease. Additionally, 7.8% had experienced either a stroke or critical lower limb ischemia requiring revascularization procedures.

At ophthalmological evaluation, DR was detected in 44 of 129 subjects (34.1%). Among these, the majority (*n* = 36) had NPDR, while eight subjects were diagnosed with PDR. The main characteristics of the study groups stratified by retinal status are reported in Table 2. No significant differences were observed between subjects with and without DR in terms of age, sex distribution, age at diabetes diagnosis, glycaemic control, or socio-demographic variables, including educational level, employment status, and smoking habits. However, subjects with DR showed a significantly longer duration of diabetes compared with those without DR (18 vs. 16 years, *p* < 0.01). This association remained significant at multivariable analysis (OR 1.12 per year increase). When stratifying according to DR severity, no significant difference in diabetes duration was observed between NPDR and PDR. A non-significant trend toward higher BMI was observed in subjects with PDR compared to NPDR and NDR (31.3 vs. 27.4 vs. 30.0 kg/m^2^, *p* = 0.09). A similar pattern was found for WC, with higher values in the PDR group, although not reaching statistical significance (Table 2). Regarding lipid profile, no differences were observed between NDR and DR groups. However, when considering DR severity, HDL cholesterol levels were significantly lower in subjects with PDR compared to NPDR and NDR (*p* = 0.01), while total cholesterol and LDL cholesterol showed significant differences across groups, with lower LDL levels in PDR compared to NPDR (*p* = 0.03). Blood pressure, triglycerides, liver enzymes, and renal function parameters did not differ significantly between groups (Table 2). With respect to pharmacological treatment, insulin therapy was significantly more frequent in subjects with DR compared to those without DR (52.3% vs. 34.1%, *p* = 0.04), with a similar trend observed across DR severity groups (Table 2). The use of other glucose-lowering agents, including GLP-1 receptor agonists and SGLT2 inhibitors, as well as lipid-lowering, antihypertensive, and antiplatelet therapies, did not differ significantly between groups.

### 3.2. Vision-Related Quality of Life Across Diabetic Retinopathy Stages

The scores of the NEI-VFQ questionnaire according to retinal status are reported in Table 3. No significant differences were observed between subjects with and without DR in the overall composite score or in most subscales. When stratified by DR severity, subjects with PDR showed lower composite scores compared with NPDR and NDR (*p* = 0.04), suggesting poorer vision-related quality of life. However, these severity-based findings should be interpreted cautiously because the PDR group included only eight participants and several NEI-VFQ domains showed high median values in the remaining groups. In addition, several specific domains were significantly impaired in subjects with PDR. In particular, general vision was reduced compared to the other groups (*p* = 0.02), and a similar pattern was observed for mental health (*p* = 0.01) and ocular pain (*p* < 0.01). Functional domains were also affected, with lower scores in near activities (*p* = 0.02), distance activities (*p* < 0.05), and driving (*p* = 0.01) (Table 3). Overall, these findings suggest that advanced DR may be associated with poorer vision-related quality of life. However, the small PDR subgroup and the marked ceiling effect of the NEI-VFQ-25 in several domains limit the strength and generalizability of this result.

### 3.3. Mediterranean Diet Adherence and Individual Score Components According to Diabetic Retinopathy Status

The median MDS was 4 (IQR 3–5) in participants with and without DR and did not differ according to DR status or across DR severity groups. Among individual MDS components, lower intake of legumes was observed in subjects with DR compared with those without DR (*p* = 0.03), whereas no other component showed a consistent difference according to DR status (Table 4).

Logistic regression models evaluating the association between legume consumption and DR are reported in Table 5. In Model 1, adjusted for age and sex, low legume consumption was associated with more than twofold higher odds of DR (OR 2.2, 95% CI 1.0–4.8, *p* = 0.042). This association remained consistent after further adjustment for diabetes duration and HbA1c in Model 2 (OR 2.3, 95% CI 1.1–5.3, *p* = 0.040). Similar results were observed in Model 3, which additionally included insulin treatment (OR 2.4, 95% CI 1.1–5.7, *p* = 0.038).

### 3.4. Exploratory Assessment of Micronutrient Intake According to Diabetic Retinopathy Status

Micronutrient intakes according to DR status and severity are reported in Table 6. These analyses were exploratory and were intended to describe potential differences in micronutrient intake beyond the predefined Mediterranean diet endpoints. No significant differences were observed between participants with and without DR for any of the evaluated micronutrients. In comparisons across DR severity groups, vitamin A and vitamin E intakes were lower among participants with PDR than among those with NPDR (*p* = 0.04 for both comparisons). However, these findings should be interpreted with caution because the PDR subgroup included only eight participants and multiple exploratory comparisons were performed without formal correction for multiplicity.

### 3.5. Exploratory Correlation Analyses of Clinical and Dietary Variables with NEI-VFQ-25 Scores

**Figure 1:**
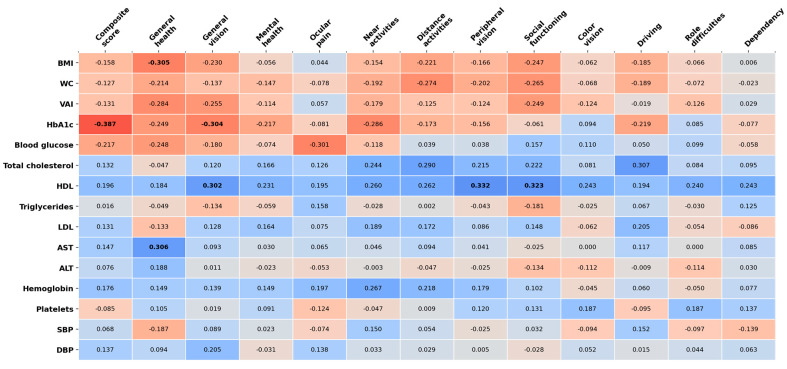
Correlation matrix between selected clinical and metabolic characteristics and NEI-VFQ-25 domain scores. Spearman correlation coefficients are shown; coefficients with *p* < 0.05 are shown in bold.

Correlation analyses between selected clinical and metabolic variables and NEI-VFQ-25 domain scores are shown in Figure 1. Overall, only a limited number of statistically significant associations were observed. HbA1c showed inverse correlations with the NEI-VFQ-25 composite score (r = −0.387) and the general vision domain (r = −0.304), suggesting lower vision-related quality-of-life scores with poorer glycaemic control. BMI was inversely correlated with general health (r = −0.305). Conversely, HDL cholesterol showed positive correlations with general vision (r = 0.302), peripheral vision (r = 0.332), and social functioning (r = 0.323). A positive correlation was also observed between AST and general health (r = 0.306).

**Figure 2:**
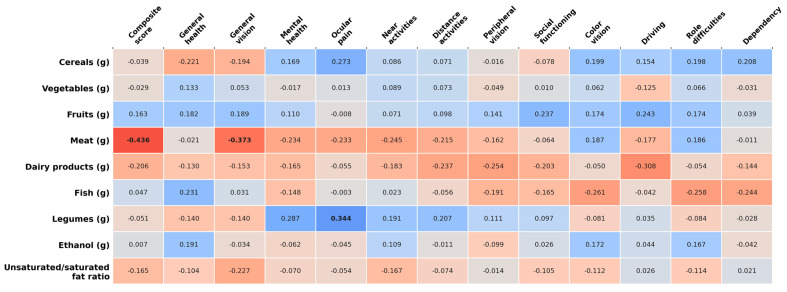
Correlation matrix between Mediterranean Diet Score components and NEI-VFQ-25 domain scores. Spearman correlation coefficients are shown; coefficients with *p* < 0.05 are shown in bold.

**Figure 3:**
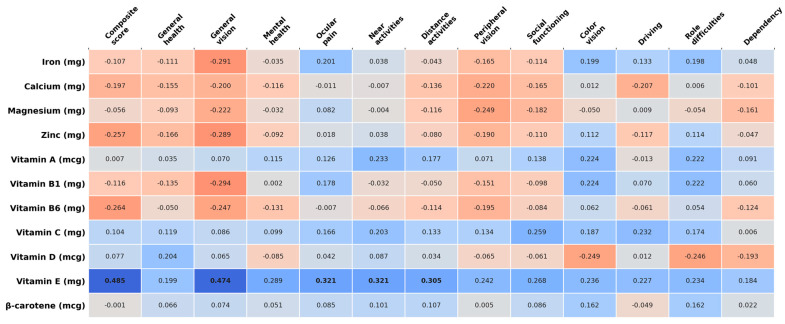
Correlation matrix between micronutrient intakes and NEI-VFQ-25 domain scores. Spearman correlation coefficients are shown; coefficients with *p* < 0.05 are shown in bold.

Correlation analyses between individual MDS components and NEI-VFQ-25 domain scores are shown in Figure 2. Among the evaluated components, meat intake was inversely correlated with the composite score and the general vision domain, whereas legume intake showed a positive correlation with the ocular pain domain. Correlation analyses between selected micronutrient intakes and NEI-VFQ-25 domain scores are reported in Figure 3. In these analyses, vitamin E showed positive correlations with several NEI-VFQ-25 domains. Given the exploratory nature of these analyses and the absence of correction for multiple testing, these findings were interpreted descriptively and should be considered hypothesis-generating.

## 4. Discussion

The present study investigated the relationships among clinical and metabolic characteristics, Mediterranean diet adherence, individual MDS components, selected micronutrient intakes, DR status, and vision-related quality of life in a cohort of subjects with type 2 diabetes. This broader analytical framework allowed us to first contextualize the role of established clinical determinants of DR and then to examine whether overall MD adherence, individual MDS components, and selected micronutrients were associated with DR and NEI-VFQ-25 scores.

Consistent with previous evidence, we confirmed the well-established role of diabetes duration as a key risk factor for DR. In our cohort, subjects with DR had a significantly longer duration of diabetes compared to those without DR, with a 12% increased risk of DR for each additional year of disease. These findings are in line with large longitudinal studies, such as the EDIC study, which reported a progressive increase in DR prevalence with disease duration, with less than 10% of subjects remaining free from DR after three decades of follow-up [48]. Similarly, predictive models have consistently identified diabetes duration as one of the strongest determinants of DR risk [49]. Nevertheless, it is noteworthy that DR may already be present at the time of diabetes diagnosis in a substantial proportion of individuals, as highlighted by a meta-analysis reporting a prevalence of approximately 15% at onset in European populations, suggesting that additional modifiable factors may contribute to the development and progression of retinal damage [50].

Among these factors, obesity and metabolic alterations have been proposed as potential contributors to DR. In our study, although BMI did not differ significantly between subjects with and without DR, a trend toward higher BMI was observed in subjects with PDR, suggesting a possible role of adiposity excess in more advanced stages of retinal disease. The relationship between obesity and DR remains controversial, with previous studies reporting conflicting results, ranging from positive associations mediated by hypertension, dyslipidaemia, oxidative stress, and vascular endothelial growth factor (VEGF) [23,51,52] to null or even inverse relationships [51,53,54]. This heterogeneity may reflect differences in study design, population characteristics, and metabolic profiles, as well as the potential influence of factors such as insulin resistance and endogenous insulin secretion.

In addition to adiposity, lipid profile may also influence the course of DR, although available evidence remains inconsistent. In our cohort, no significant differences were observed in total cholesterol, LDL cholesterol, or triglycerides between subjects with and without DR. However, when stratifying by disease severity, subjects with PDR showed significantly lower HDL cholesterol levels compared to those with NPDR and no DR. The role of HDL cholesterol in DR is not fully understood. While some studies have reported no association between lipid levels and DR [55], others have suggested a complex or even paradoxical relationship, including a potential U-shaped association between HDL levels and DR risk [56,57]. These findings indicate that lipid metabolism may play a nuanced role in retinal microvascular damage, warranting further investigation.

Pharmacological treatment patterns also differed according to DR severity. Insulin therapy was more frequently observed in subjects with DR, with the highest prevalence in PDR [58,59]. This observation is likely to reflect longer disease duration and greater diabetes severity rather than a direct causal effect [60]. The lower use of metformin observed among subjects with DR should also be interpreted cautiously, because the present study was not designed to evaluate independent medication effects on DR [61,62,63].

The evaluation of vision-related quality of life represents a relevant and often underexplored aspect of DR [64,65,66]. In our study, no significant differences were observed between subjects with and without DR overall, whereas lower NEI-VFQ scores were observed among subjects with PDR. This finding is clinically plausible but should be interpreted as exploratory because only eight participants had PDR and most questionnaire domains showed a ceiling effect, with median scores at or near the maximum in NDR and NPDR groups. In addition, residual confounding by visual acuity, macular oedema, cataract status, or previous retinal treatment cannot be excluded unless these variables are specifically considered in future analyses.

A central aspect of the present study was the evaluation of dietary habits within a prespecified analytical hierarchy, in line with the broader evidence suggesting a potential role of diet quality in the development and progression of DR [29,67,68]. Overall MD adherence, assessed by the MDS, did not differ between participants with and without DR, indicating a null finding for the primary dietary endpoint. When individual MDS components were examined as secondary endpoints, however, low legume consumption was associated with the presence of DR. This association was observed in multivariable models including established clinical determinants of DR, such as diabetes duration and HbA1c [8,9,21]. Insulin treatment was additionally evaluated to account for treatment intensity and the broader clinical severity of diabetes, while limiting the risk of overfitting.

This association is biologically plausible because legumes are low-glycaemic-index, fibre-rich foods that may improve carbohydrate quality and cardiometabolic risk factors relevant to microvascular disease [69,70,71,72,73]. However, the present cross-sectional study cannot establish whether low legume intake preceded DR, resulted from dietary changes after DR diagnosis, or reflects residual confounding. Accordingly, the legume finding should be regarded as hypothesis-generating and not as evidence that legumes prevent or treat DR. Exploratory findings involving vitamin E and other micronutrients may reflect antioxidant and retinal-health pathways, but these results require confirmation after accounting for multiple comparisons and in larger prospective cohorts.

Several limitations should be considered when interpreting these findings. First, the cross-sectional design precludes causal or temporal inference. Therefore, it cannot be determined whether the observed dietary differences preceded the development of DR, reflected dietary changes after DR diagnosis, or were explained by residual or unmeasured confounding. This is particularly relevant for legume consumption, as reverse causation cannot be excluded. Second, dietary intake was assessed using an FFQ referring to the previous month. Although this approach allowed standardized assessment of habitual intake close to the time of ophthalmological evaluation, it may not adequately capture long-term dietary exposure relevant to the development or progression of DR, which usually occurs over many years. In addition, FFQ-based dietary assessment is subject to recall error, portion-size misclassification, and measurement error in nutrient estimation. Third, the sample size was limited, particularly for severity-based analyses. Only a small number of participants had PDR, making comparisons involving this subgroup unstable and exploratory. For this reason, findings related to DR severity, including those concerning NEI-VFQ-25 scores and micronutrient intake in the PDR group, should be interpreted cautiously. Fourth, the NEI-VFQ-25 showed a marked ceiling effect in several domains, with many participants achieving very high scores. This may have limited the ability of the questionnaire to discriminate differences in vision-related quality of life among participants with relatively preserved visual function and may have reduced the robustness of correlation analyses involving NEI-VFQ-25 domains. Fifth, multiple comparisons were performed, particularly in analyses of micronutrient intake and correlation matrices. Although these analyses were explicitly considered exploratory, no formal correction for multiplicity was applied. Therefore, the possibility of false-positive findings cannot be excluded, and these results should be interpreted as hypothesis-generating rather than confirmatory. Sixth, although multivariable models were deliberately kept parsimonious to reduce the risk of overfitting, the number of DR cases was limited. This constrained the number of covariates that could be included and may have resulted in residual confounding. Conversely, adding a larger number of clinical, metabolic, pharmacological, and lifestyle variables would have reduced model stability. The selected models therefore represent a compromise between adjustment for key a priori confounders and preservation of statistical reliability. Seventh, the study population was selected according to specific eligibility criteria, including long-standing T2D and a restricted HbA1c range. While this design reduced clinical heterogeneity, it may limit the generalizability of the findings to individuals with shorter diabetes duration, very good glycaemic control, poor glycaemic control, or different demographic and clinical characteristics. The restricted variability in diabetes duration and HbA1c may also have attenuated associations with established predictors of DR. Eighth, although the ophthalmological assessment was comprehensive, some factors that may influence vision-related quality of life, such as cataract severity, diabetic macular edema, previous retinal treatments, and other subtle ocular comorbidities, were not fully accounted for in the statistical analyses. These factors may have affected NEI-VFQ-25 scores independently of DR stage. Finally, residual confounding from unmeasured or incompletely measured variables cannot be excluded. These include physical activity, supplement use, socioeconomic characteristics, health literacy, access to care, adherence to diabetes treatment, overall diet quality beyond the MDS, and long-term changes in dietary habits. Accordingly, the present findings should be interpreted as exploratory and require confirmation in larger prospective studies with repeated dietary assessments and adequate power for DR severity subgroups.

At the same time, the study also has several strengths. First, DR status was defined through a comprehensive ophthalmological evaluation, including multimodal retinal assessment, and retinal findings were independently reviewed by two experienced ophthalmologists. This approach reduced the risk of outcome misclassification and strengthened the clinical characterization of DR status and severity. Second, the study integrated objective clinical classification of DR with patient-reported vision-related quality of life assessed using the NEI-VFQ-25. This allowed the burden of DR to be evaluated from both a clinical and patient-centered perspective, providing information not only on retinal disease status but also on its perceived functional impact. Third, the study focused on individuals with long-standing T2D, a population at increased risk of DR and therefore clinically relevant for investigating potential modifiable factors associated with retinal complications. Although this selection limits generalizability, it also reduced heterogeneity related to diabetes duration and allowed the analyses to be conducted in a group with substantial cumulative exposure to diabetes. Fourth, dietary assessment was structured around a clear analytical hierarchy. Overall adherence to MD was evaluated as the primary dietary endpoint, individual MDS components were assessed as secondary endpoints, and micronutrient and correlation analyses were explicitly treated as exploratory. This framework improves transparency and reduces the risk of overinterpreting secondary or exploratory findings.

## 5. Conclusions

In this cross-sectional study of individuals with long-standing T2D, overall adherence to MD, assessed using the MDS, was not associated with the presence of DR. This null finding for the primary dietary endpoint suggests that, in this selected cohort, global adherence to a Mediterranean dietary pattern did not clearly distinguish participants with DR from those without DR. In secondary analyses focused on individual components of the MDS, low legume consumption was associated with higher odds of DR in multivariable models. This association persisted after adjustment for key a priori selected clinical determinants, including diabetes duration and HbA1c, and after additional consideration of insulin treatment as a marker of diabetes severity and treatment intensity. However, given the observational and cross-sectional design, this finding should be interpreted as an association rather than evidence of a protective effect of legumes against DR. Exploratory analyses further suggested that selected micronutrients, particularly vitamin E, may be associated with vision-related quality-of-life domains, while advanced DR was associated with poorer NEI-VFQ-25 scores. Nevertheless, these findings require cautious interpretation because of the small number of participants with proliferative DR, the ceiling effect observed in several NEI-VFQ-25 domains, and the absence of formal correction for multiple exploratory comparisons. Overall, the present findings support the hypothesis that specific dietary components may be more informative than global dietary scores in relation to DR status and patient-reported visual function. However, they do not establish causality and should not be used to formulate clinical dietary recommendations for DR management at this stage. Larger prospective studies, ideally including repeated dietary assessments, broader glycaemic and clinical profiles, and adequately powered DR severity subgroups, are needed to confirm these observations and to clarify whether specific dietary components may contribute to the prevention or progression of diabetic retinal disease.

## Figures and Tables

**Table 1:** Demographic, anthropometric, clinical and biochemical characteristics of the 129 recruited subjects.

Characteristics	Median (IQR) or *n* (%)
Male gender, *n* (%)	65 (50.4)
Age (years)	70 (65–74)
Duration of diabetes (years)	17 (14–19)
BMI (kg/m^2^)	29.8 (26.3–33.8)
BMI classification, *n* (%)	
Normal weight	17 (13.2)
Overweight	54 (41.9)
Obese	58 (45.0)
WC (cm)	105 (98–115)
High WC, *n* (%)	104 (80.6)
HbA1c (%)	8 (7–8)
Fasting plasma glucose (mg/dL)	133 (121–158)
Creatinine (mg/dL)	1 (1–1)
eGFR (mL/min)	77.5 (58.4–91.4)
Microalbuminuria, *n* (%)	24 (18.6)
Macroalbuminuria, *n* (%)	1 (0.8)
Cholesterol total (mg/dL)	164 (143–182)
HDL (mg/dL)	46 (39–53)
LDL (mg/dL)	90 (70–109)
Triglycerides (mg/dL)	117 (89–151)
AST (U/L)	18 (16–23)
ALT (U/L)	19 (14–28)
SBP (mmHg)	130 (120–145)
DBP (mmHg)	75 (70–80)
Insulin treatment, *n* (%)	54 (41.9)
Long-acting insulin analogues	54 (41.9)
Short-acting insulin analogues	19 (14.7)
Other diabetes drugs, *n* (%)	
Metformin	111 (86)
GLP-1 RA	59 (45.7)
SGLT2-I	31 (24)
GLP-1 RA or SGLT2-I	81 (62.8)
DPP4-I	13 (10.2)
Pioglitazone	17 (13.2)
Acarbose	3 (2.3)
Lipid lowering drugs, *n* (%)	99 (76.7)
Anti-hypertensive drugs, *n* (%)	106 (82.2)
Anti-platelets drugs, *n* (%)	89 (71.2)
Ischemic heart disease	21 (16.3)
Stroke or lower limb revascularization	10 (7.8)

BMI, body mass index; WC, waist circumference; eGFR, estimated glomerular filtration rate; SBP, systolic blood pressure, DBP, diastolic blood pressure; GLP-1 RA, glucagon like peptide 1 receptor agonists; SGLT2-I, sodium glucose transporter-2 inhibitors; DPP4-I, dipeptidyl peptidase-4 inhibitors.

**Table 2:** Clinical and biochemical characteristics of the study population according to retinal status.

	NDR (*N* = 85)	DR (*N* = 44)	NPDR (*N* = 36)	PDR (*N* = 8)	*p* (NDR vs. DR)	*p* (NDR vs. NPDR vs. PDR)	*p* (NPDR vs. PDR)
Male gender, *n* (%)	38 (44.7)	27 (61.4)	26 (63.9)	4 (50.0)	0.07	0.16	0.47
Age (years)	70 (63.5–74)	70 (65.3–76)	71 (66–76)	70 (64–75)	0.46	0.65	0.56
Age at diabetes diagnosis (years)	53 (47–58)	53 (45–56)	54 (45–56)	51 (40–59)	0.31	0.57	0.62
Duration of diabetes (years)	16 (13–19)	18 (16–21.8)	18 (15–22)	18 (17–21)	<0.01	<0.01	0.78
BMI (kg/m^2^)	30 (26.4–34.3)	28.1 (25.6–32.7)	27.4 (26–32)	31.3 (26–39)	0.12	0.09	0.17
BMI classification, *n* (%)							
Normal weight	10 (11.8)	7 (15.9)	6 (16.7)	1 (12.5)	0.20	0.38	0.58
Overweight	32 (37.6)	22 (50.0)	19 (52.8)	3 (37.5)			
Obese	43 (50.6)	15 (34.1)	11 (30.6)	4 (50)			
WC (cm)	105 (97–115)	107 (101–114.8)	106 (103–112)	115 (105–125)	0.53	0.29	0.13
High WC, *n* (%)	69 (81.2)	35 (79.5)	28 (77.8)	7 (87.5)	0.82	0.80	0.54
HbA1c (%)	7 (7–8)	8 (7–8)	8 (7–8)	8 (7–8)	0.92	0.23	0.80
Fasting glucose (mg/dL)	133 (121–156)	132 (121–161)	132 (121–161)	131 (119–171)	0.67	0.91	0.96
Creatinine (mg/dL)	1 (1–1)	1 (1–1)	1 (1–1)	1 (1–1)	0.70	0.83	0.08
GFR (mL/min)	79.8 (61.5–92)	74.7 (57–87)	71.4 (56.8–87)	82.0 (72–96)	0.41	0.34	0.27
Microalbuminuria, *n* (%)	15 (17.6)	9 (20.5)	6 (16.7)	3 (37.5)	0.71	0.36	0.19
Macroalbuminuria, *n* (%)	1 (1.2)	0 (0.0)	0 (0.0)	0 (0.0)	-	-	-
Cholesterol total (mg/dL)	162 (141.3–179)	166 (144–187)	169 (153–192)	145 (120–152)	0.56	0.04	0.01
HDL (mg/dL)	45 (38–52.8)	49 (41–54)	50 (43–59)	40 (33–46)	0.17	0.01	0.01
LDL (mg/dL)	87.5 (66.3–109)	90 (73–108)	95 (80–112)	78 (61–84)	0.95	0.16	0.03
Triglycerides (mg/dL)	123 (89–151)	110 (84.3–163.5)	107 (84–164)	127 (80–163)	0.56	0.80	0.89
AST (U/L)	19 (16–24)	18 (14–21)	18 (14–21)	18 (16–21)	0.16	0.37	0.96
ALT (U/L)	20 (14–29)	17 (14–24)	17 (13–24)	21 (16–33)	0.19	0.26	0.31
SBP (mmHg)	130 (123–145)	133 (120–144)	133 (125–144)	128 (120–144)	0.91	0.83	0.58
DBP (mmHg)	75 (70–80)	70 (66–80)	70 (66–80)	70 (66–78)	0.10	0.20	0.54
Insulin treatment, *n* (%)	29 (34.1)	23 (52.3)	17 (47.2)	6 (75.0)	0.04	0.03	0.20
Long-acting insulin analogues	29 (34.1)	23 (52.3)	17 (47.2)	6 (75.0)	<0.05	<0.05	0.20
Short-acting insulin analogues	8 (9.4)	11 (25.0)	9 (25.0)	2 (25.0)	0.02	0.06	1.00
Pro-kg insulin daily dose (U)							
Long-acting insulin analogues	0.26 (0.18–0.38)	0.27 (0.17–0.37)	0.24 (0.16–0.30)	0.4 (0.3–0.5)	0.96	0.07	0.02
Short-acting insulin analogues	0.26 (0.17–0.57)	0.26 (0.21–0.32)	0.24 (0.18–0.37)	0.27 (0.26–NA)	0.60	0.76	0.73
Total dose	0.29 (0.20–0.46)	0.39 (0.22–0.56)	0.36 (0.24–0.52)	0.51 (0.34–0.62)	0.23	0.27	0.25
Other diabetes drugs, *n* (%)							
Metformin	78 (91.8)	33 (75.0)	26 (72.2)	7 (87.5)	<0.01	0.02	0.37
GLP-1 RA	38 (44.7)	21 (47.7)	16 (44.4)	5 (62.5)	0.74	0.62	0.36
SGLT2-I	16 (18.8)	15 (34.1)	13 (36.1)	2 (25.0)	0.054	0.13	0.55
GLP-1 RA or SGLT2-I	47 (55.3)	34 (77.3)	28 (77.8)	6 (75.0)	0.01	<0.05	0.87
DPP4-I	11 (13.4)	2 (4.5)	2 (5.6)	0 (0.0)	0.13	0.28	0.50
Pioglitazone	11 (12.9)	6 (13.6)	6 (16.7)	0 (0.0)	0.91	0.45	0.21
Acarbose	3 (3.5)	0 (0.0)	0 (0.0)	0 (0.0)	0.21	0.45	-
Lipid lowering drugs, *n* (%)	68 (80.0)	31 (70.5)	25 (69.4)	6 (75.0)	0.22	0.45	0.76
Anti-hypertensive drugs, *n* (%)	72 (84.7)	34 (77.3)	28 (77.8)	6 (75.0)	0.30	0.57	0.87
Anti-platelets drugs, *n* (%)	56 (69.1)	33 (75)	28 (77.8)	5 (62.5)	0.49	0.54	0.37

NDR, no diabetic retinopathy; DR: diabetic retinopathy, NPDR, non-proliferant diabetic retinopathy; PDR, proliferant diabetic retinopathy; BMI, body mass index; WC, waist circumference; GFR, glomerular filtration rate; SBP, systolic blood pressure, DBP, diastolic blood pressure; GLP-1 RA, glucagon like peptide 1 receptor agonists; SGLT2-I, sodium glucose transporter-2 inhibitors; DPP4-I, dipeptidyl peptidase-4 inhibitors.

**Table 3:** Scores of the National Eye Institute Visual Function Questionnaire in the recruited subjects subdivided according to retinal status.

	NDR (*N* = 85)	DR (*N* = 44)	NPDR (*N* = 36)	PDR (*N* = 8)	*p* (NDR vs. DR)	*p* (NDR vs. NPDR vs. PDR)	*p* (NPDR vs. PDR)
COMPOSITE SCORE	99.1 (95.9–99.5)	99.1 (97–99)	99.1 (97.6–99.1)	94.6 (85.7–98)	0.47	0.04	0.01
General health	70.0 (60–77.5)	65.0 (60.0–77.5)	65 (60–75)	72.5 (60–77.5)	0.63	0.92	0.96
General vision	90.0 (70–95)	87.5 (75–90)	90 (82.5–90)	72.5 (55–85)	0.35	0.07	0.02
Mental health	100 (95–100)	100 (100–100)	100 (100–100)	98 (83–100)	0.17	0.01	0.07
Ocular pain	100 (88–100)	100 (100–100)	100 (100–100)	88 (82–100)	0.16	<0.01	0.02
Near activities	100 (100–100)	100 (100–100)	100 (100–100)	96 (63–100)	0.62	0.02	0.07
Distance activities	100 (100–100)	100 (100–100)	100 (100–100)	100 (81–100)	0.44	<0.05	0.16
Peripheral vision	100 (100–100)	100 (100–100)	100 (100–100)	100 (88–100)	0.69	0.17	0.40
Social functioning	100 (100–100)	100 (100–100)	100 (100–100)	100 (96–100)	0.77	0.10	0.33
Color vision	100 (100–100)	100 (100–100)	100 (100–100)	100 (100–100)	0.28	0.19	0.59
Driving	100 (100–100)	100 (100–100)	100 (100–100)	96 (67–100)	0.74	0.01	0.11
Role difficulties	100 (100–100)	100 (100–100)	100 (100–100)	100 (100–100)	0.15	0.14	0.60
Dependency	100 (100–100)	100 (100–100)	100 (100–100)	100 (100–100)	0.44	0.42	0.67

NDR, no diabetic retinopathy; DR: diabetic retinopathy, NPDR, non-proliferant diabetic retinopathy; PDR, proliferant diabetic retinopathy.

**Table 4:** Mediterranean Diet Score and individual score components by diabetic retinopathy status.

Variable	NDR (*N* = 85)	DR (*N* = 44)	NPDR (*N* = 36)	PDR (*N* = 8)	*p* (NDR vs. DR)	*p* (NDR vs. NPDR vs. PDR)	*p* (NPDR vs. PDR)
MDS	4 (3–5)	4 (3–5)	4 (3–5)	4 (3–5)	0.66	0.52	0.63
Cereals (g)	168.3 (113.5–206.6)	180.8 (136.7–218.5)	186.7 (141.9–223.5)	168.9 (126.8–182.1)	0.19	0.21	0.26
Vegetables (g)	345.7 (226.7–478.2)	331.0 (258.3–416.1)	331.0 (251.5–424.0)	329.0 (263.7–361.9)	0.60	0.76	0.62
Fruits (g)	310.0 (193.3–377.3)	231.2 (191.2–321.2)	240.5 (193.6–357.6)	212.3 (187.5–231.9)	0.15	0.15	0.23
Meat (g)	61.3 (48.0–80.0)	59.5 (48.5–75.0)	57.2 (48.5–75.0)	60.7 (56.2–75.5)	0.94	0.96	0.71
Dairy products (g)	278.7 (103.8–331.7)	269.2 (98.0–329.0)	208.5 (88.4–329.0)	315.8 (247.1–333.9)	0.96	0.69	0.50
Fish (g)	53.9 (26.9–86.9)	51.4 (34.7–74.2)	51.4 (39.7–66.4)	41.9 (25.2–86.1)	0.60	0.84	0.73
Legumes (g)	40.3 (16.7–50.6)	34.0 (21.8–53.1)	32.2 (23.5–53.6)	36.7 (27.6–47.7)	0.03	0.08	0.33
Ethanol (g)	0 (0–0.1)	0.1 (0–0.1)	0.1 (0–0.1)	0.1 (0–0.1)	0.20	0.30	0.13
Unsaturated/Saturated fat ratio	2.42 (1.98–2.74)	2.30 (1.96–2.93)	2.26 (1.89–2.80)	2.71 (2.31–2.93)	0.54	0.19	0.14

NDR, no diabetic retinopathy; DR, diabetic retinopathy; NPDR, non-proliferative diabetic retinopathy; PDR, proliferative diabetic retinopathy; MDS, Mediterranean Diet Score. Values are presented as median (interquartile range).

**Table 5:** Logistic regression models for the association between low legume consumption and the odds of diabetic retinopathy.

Model	Comparison	OR (95%CI)	*p*-Value
Model 1	Legumes (low vs. high consumption)	2.2 (1.0–4.8)	0.042
Model 2	Legumes (low vs. high consumption)	2.3 (1.1–5.3)	0.040
Model 3	Legumes (low vs. high consumption)	2.4 (1.1–5.7)	0.038

Model 1: age and sex. Model 2: age, sex, diabetes duration, and HbA1c. Model 3: Model 2 plus insulin treatment. Low legume consumption was defined as intake below or equal to the cohort-specific median.

**Table 6:** Micronutrient intakes by diabetic retinopathy status.

Variable	NDR (*N* = 85)	DR (*N* = 44)	NPDR (*N* = 36)	PDR (*N* = 8)	*p* (NDR vs. DR)	*p* (NDR vs. NPDR vs. PDR)	*p* (NPDR vs. PDR)
Iron (mg)	10.8 (9.2–12.6)	10.8 (9.7–13.2)	11.2 (9.8–13.2)	9.8 (8.4–10.9)	0.56	0.21	0.11
Calcium (mg)	914.9 (722.0–1100.4)	941.3 (725.9–1244.5)	966.0 (736.6–1244.5)	867.8 (630.6–1125.7)	0.32	0.43	0.50
Magnesium (mg)	297.8 (245.5–339.5)	299.2 (256.0–329.0)	303.2 (267.2–342.6)	293.8 (244.5–310.8)	0.68	0.42	0.22
Zinc (mg)	8.6 (6.6–9.6)	8.6 (7.2–10.5)	8.7 (8.0–10.5)	7.5 (6.0–9.1)	0.41	0.25	0.15
Vitamin A (mcg)	891.9 (579.8–1148.3)	869.9 (687.4–1096.0)	889.4 (708.3–1113.1)	728.4 (505.2–834.9)	0.93	0.14	0.04
Vitamin B1 (mg)	1.23 (1.07–1.46)	1.29 (1.13–1.47)	1.28 (1.14–1.47)	1.29 (0.96–1.43)	0.37	0.50	0.50
Vitamin B6 (mg)	1.68 (1.31–1.91)	1.62 (1.36–1.85)	1.63 (1.42–1.88)	1.40 (1.27–1.63)	0.68	0.28	0.08
Vitamin C (mg)	118.5 (82.3–156.8)	118.7 (87.8–142.6)	123.1 (100.7–145.9)	92.3 (79.3–120.7)	0.88	0.63	0.27
Vitamin D (mcg)	5.1 (2.9–8.6)	5.0 (4.1–7.6)	5.1 (4.2–7.6)	4.5 (1.6–5.9)	0.70	0.60	0.30
Vitamin E (mg)	28.5 (25.0–40.1)	27.3 (21.4–34.2)	37.2 (30.2–46.6)	26.8 (20.0–33.4)	0.45	0.51	0.04
β-carotene (µg)	7303.4 (5430.2–9893.6)	6094.9 (4560.4–8039.8)	6199.1 (3560.6–7305.6)	5484.7 (4019.5–7824.5)	0.54	0.45	0.19

NDR, no diabetic retinopathy; DR, diabetic retinopathy; NPDR, non-proliferative diabetic retinopathy; PDR, proliferative diabetic retinopathy. Values are presented as median (interquartile range).

## Data Availability

Data used to support the findings of this study are available from the corresponding author upon request.
